# Supplementary material for: The genetic architecture of multimodal human brain age
Source: Nat Commun. 2024 Mar 23;15:2604. doi: 10.1038/s41467-024-46796-6 (PMC10960798; doi:10.1038/s41467-024-46796-6)
Supplement: Supplementary file 1 — Supplementary Information [file 41467_2024_46796_MOESM1_ESM.pdf]

The Genetic Architecture of Multimodal Human Brain Age

Junhao Wen[1*], Bingxin Zhao[2], Zhijian Yang[3], Guray Erus[3], Ioanna Skampardoni[3], Elizabeth Mamourian[3], Yuhan Cui[3], Gyujoon Hwang[3], Jingxuan Bao[4], Aleix Boquet-Pujadas[5], Zhen Zhou[3], Yogasudha Veturi[6], Marylyn D. Ritchie[7], Haochang Shou[3], Paul M. Thompson[8], Li Shen[4], Arthur W. Toga[9], Christos Davatzikos[3]

[1]Laboratory of AI and Biomedical Science (LABS), Stevens Neuroimaging and Informatics Institute, Keck School of Medicine of USC, University of Southern California, Los Angeles, CA, USA
[2]Department of Statistics and Data Science, University of Pennsylvania, Philadelphia, PA, USA
[3]Artificial Intelligence in Biomedical Imaging Laboratory (AIBIL), Center for AI and Data Science for Integrated Diagnostics (AI^{2}D), Perelman School of Medicine, University of Pennsylvania, Philadelphia, PA, USA
[4]Department of Biostatistics, Epidemiology and Informatics, University of Pennsylvania Perelman School of Medicine, Philadelphia, PA, USA
[5]Biomedical Imaging Group, EPFL, Lausanne, Switzerland
[6]Department of Biobehavioral Health and Statistics, Penn State University, University Park, PA, USA
[7]Department of Genetics and Institute for Biomedical Informatics, Perelman School of Medicine, University of Pennsylvania, Philadelphia, PA, USA
[8]Imaging Genetics Center, Mark and Mary Stevens Neuroimaging and Informatics Institute, Keck School of Medicine of USC, University of Southern California, Marina del Rey, CA, USA
[9]Laboratory of Neuro Imaging (LONI), Stevens Neuroimaging and Informatics Institute, Keck School of Medicine of USC, University of Southern California, Los Angeles, CA, USA

[*]Corresponding author:
Junhao Wen, junhaowe@usc.edu
2025 Zonal Ave, Los Angeles, CA 90033, United States

Supplementary method 1: Image quality check for MUSE

Supplementary method 2: The definition of genomic loci, independent significant SNP, lead SNP, candidate SNP

Supplementary note1: Seven sensitivity check analyses for the three primary GWASs on European ancestry populations

Supplementary note2: Exemplary genomic locus linked to GM, WM, and FC-BAG

Supplementary figure 1: Split-sample genome-wide association results

Supplementary figure 2: Sex-stratified genome-wide association results

Supplementary figure 3: Non-European genome-wide association results

Supplementary figure 4: fastGWA for mixed linear models

Supplementary figure 5: Machine learning-specific GWAS

Supplementary figure 6: Feature type-specific GWAS

Supplementary figure 7: Independent WGS dataset for GM-BAG GWAS

Supplementary figure 8: Genetic correlation (g_c) between the GM, WM, and FC-BAG using the LDSC software in the split-sample analyses

Supplementary figure 9: Incremental R2 of the PRS derived by the PLINK C+T approach

Supplemenatry figure 10: Results for the inverse Mendelian randomization for the seven clinical traits

Supplementary figure 11: Sensitivity check for all other significant exposure variables in the forward MR analyses for A) cancer on GM-BAG, B) diabetes on GM-BAG, and C) AD on WM-BAG

Supplementary figure 12: RNA expression overview of the *DNAJC1* gene in various cancer types

Supplementary figure 13: The five-layer neural network used for age prediction and its performance using WM-IDP

Supplementary table 1: Brain age prediction performance using GM, WM, and FC-IDP

Supplementary table 2: Identified genomic loci and mapped genes

Supplementary table 3: Selected clinical traits for genetic correlation analyses

Supplementary table 4: Selected exposure variables for the forward Mendelian randomization

Supplementary table 5: Study characteristics.

Supplementary method 1: Image quality check for MUSE

T1-weighted MRIs were first quality checked (QC) for motion, image artifacts, or restricted field-of-view. Additional QC was performed as follows: First, the images were examined by manually evaluating for pipeline failures (e.g., poor brain extraction, tissue segmentation, and registration errors). Furthermore, a second step automatically flagged images based on outlying values of quantified metrics; those images were re-evaluated.

Supplementary method 2: The definition of genomic loci, independent significant SNP, lead SNP, candidate SNP

FUMA defined the significant independent SNPs, lead SNPs, candidate SNPs, and genomic risk loci as follows (https://fuma.ctglab.nl/tutorial#snp2gene):

Independent significant SNPs

They are defined as SNPs with $P \leq 5 \times 10^{-8}$ independent of each other at the user-defined r^2 (set to 0.6 in the current study). We further describe *candidate SNPs* as those in linkage disequilibrium (LD) with independent significant SNPs. FUMA then queries each candidate SNP in the GWAS Catalog to check whether any clinical traits have been reported to be associated with previous GWAS studies.

Lead SNPs

Lead SNPs are defined as independent significant SNPs that are also independent of each other at $r^2<0.1$. If multiple independent significant SNPs are correlated at $r^2 \geq 0.1$, then the one with the lowest individual *P*-value becomes the lead SNP. If the r^2 threshold is set to 0.1 for the independent significant SNPs, then they will constitute the identical set as the lead SNPs by definition. FUMA thus advises setting r^2 to be 0.6 or higher.

Genomic risk loci

FUMA defines genomic risk loci to include all independent signals physically close or overlapping in a single locus. First, independent significant SNPs dependent on each other at $r^2 \geq 0.1$ are assigned to the same genomic risk locus. Then, independent significant SNPs with less than the user-defined distance (250 kilobases by default) away from one another are merged into the same genomic risk locus - the distance between two LD blocks of two independent significant SNPs is the distance between the closest points from each LD block. Each locus is represented by the SNP within the locus with the lowest *P*-value.

Supplementary note1: Seven sensitivity check analyses for the three primary GWASs on European ancestry populations

We performed seven sensitivity check analyses to scrutinize the robustness of our primary GWASs on European ancestry populations.

Split-sample GWAS

P-value:

We noted high concordance rates between the split1 (as discovery, $15{,}778<N<16{,}008$) and split2 (as replication, $15{,}778<N<16{,}008$) GWASs. Specifically, for GM-BAG, we observed a concordance rate of 99% (3090 out of 3092 SNPs; P-value<0.05/3092), and for WM-BAG, the concordance rate reached 100% (116/116). FC-BAG did not achieve significant genome-wide results in the split-sample GWASs

β value:

We assessed the concordance of the β values between split1 and split2 GWASs. For GM-BAG, all the 3092 significantly replicated SNP (P-value<0.05) showed the same sign of β values from the linear regression models (Pearson's r=0.67; P-value<1x10^{-10}). For WM-BAG, all the 116 significantly replicated SNP (P-valure<0.05) showed the same sign (Pearson's r=0.98; P-value<1x10^{-10}) (**Supplementary Figure 1** and **eFile 1**).

Sex-stratified GWAS

P-value:

In sex-stratified GWASs, the concordance rates were 100% (3072/3072, P-value<0.05/3072) for GM-BAG and 88.6% (116/131, P-value<0.05/131) for WM-BAG when comparing the male-GWAS (as replication, $14{,}969<N<15{,}127$) to female-GWAS (as discovery, $16{,}588<N<16{,}890$). FC-BAG did not achieve significant genome-wide results.

β value:

For GM-BAG, the 3072 significantly replicated SNP (P-valure<0.05) showed the same sign of *β* values from the linear regression models (Pearson's *r*=0.36; P-value<$1x10^{-10}$). For WM-BAG, the 116 significantly replicated SNP (P-valure<0.05) showed the same sign (Pearson's *r*=0.99; P-value<$1x10^{-10}$) (**Supplementary Figure 2** and **eFile 2**).

Non-European GWAS

P-value:

The concordance rates of the GWASs using non-European ancestry populations (as replication, 4646<*N*<5091) were low compared to the main GWASs using the European population: only 13.78% for GM-BAG (277/2009; P-value<0.05) and 41.94% for WM-BAG (198/472; P-value<0.05).

β value:

For GM-BAG, the 277 significantly replicated SNP (P-valure<0.05) showed the same sign of *β* values from the linear regression models (Pearson's *r*=0.97; P-value<$1x10^{-10}$). For WM-BAG, the 198 significantly replicated SNP (P-valure<0.05) showed the same sign (Pearson's *r*=0.99; P-value<$1x10^{-10}$) (**Supplementary Figure 3** and **eFile 3**)

Mixed linear model GWAS

P-value:

A mixed linear model employed via fastGWA[1] (as replication, 31,557<*N*<32,017) obtained 100% concordance rates for GM (3382/3382), WM (521/521), and FC-BAG (2/2) compared to GWAS using PLINK linear regression. The genetic loci, genomic inflation factor (λ), and the

LDSC intercepts for GM, WM, and FC-BAG were similar between the PLINK and fastGWA analyses.

β value:

For GM-BAG, the 3382 significantly replicated SNP (P-valure<0.05) showed the same sign of *β* values from the linear regression models (Pearson's *r*=1; P-value<$1x10^{-10}$). For WM-BAG, the 521significantly replicated SNP (P-valure<0.05) showed the same sign (Pearson's *r*=1; P-value<$1x10^{-10}$). For FC-BAG, the 2 significantly replicated SNP (P-valure<0.05) showed the same sign. (**Supplementary Figure 4** and **eFile 4**).

Machine learning model-specific GWAS

We used GM-BAG to demonstrate this sensitivity check by comparing *i*) SVR using MUSE ROIs and *ii*) CNN using voxel images[2] (GWAS summary statistics shared by the authors) to our main results obtained from Lasso using MUSE ROIs.

P-value:

When comparing the SVR using MUSE ROIs (as replication, MAE=4.43 years) to Lasso using MUSE ROIs (as discovery, MAE=4.39 years), we found a 100% concordance rate of the SNPs identified for the GM-BAG GWAS. The BAGs derived from the two machine learning models were highly correlated (r=0.99; P-value<$1x10^{-10}$).

When comparing the CNN using voxel-wise images (MAE~2.5 years[2]) to Lasso using MUSE ROIs (as discovery), we found an 82.70% concordance rate (2533; 319 missing SNPs) after Bonferroni correction (P-value<0.05/3063).

β value:

When comparing the SVR using MUSE ROIs to Lasso using MUSE ROIs (as discovery), we found that the 3382 significantly replicated SNP (P-value<0.05) showed the same sign of β values from the linear regression models (Pearson's r=1; P-value<$1x10^{-10}$).

When comparing the CNN using voxel-wise images (MAE~2.5 years[2]) to Lasso using MUSE ROIs (as discovery), we found that all 2762 significantly replicated SNP (P-valure<0.05) showed the same sign of β values from the linear regression models (Pearson's r=1; P-value<$1x10^{-10}$). (**Supplementary Figure 5** and **eFile 5**).

Feature type-specific GWAS

P-value:

We finally found a 92.43% concordance rate of the SNPs identified in the GM-BAG GWAS using the 119 MUSE ROIs[3] (as discovery, MAE=4.39 years) and voxel-wide RAVENS[4] maps (as replication, P-value < 0.05/3382, MAE=5.12 years). The BAGs derived from the two types of features were significantly correlated (r=0.74; P-value<$1x10^{-10}$). The brain age prediction performance using RAVENS showed marginal overfitting, with an MAE of 4.31 years in the training/validation/test dataset and an MAE of 5.12 years in the independent test dataset.

β value:

3183 significantly replicated SNP (P-valure<0.05) showed the same sign of β values from the linear regression models (Pearson's r=0.99; P-value<$1x10^{-10}$) (**Supplementary Figure 6** and **eFile 6**)

ADNI WGS GWAS

P-value:

We evaluated the generalizability of the GM-BAG GWAS findings from the UKBB dataset to the ADNI whole-genome sequencing (WGS) data. When considering the concordance rate based on P-values, we observed a high concordance rate (83.57 %) for the GWASs performed using the ADNI WGS data (*N*=1104) as a replication dataset (*N*=2583 out of 3091; 291 SNPs missing from the ADNI data) using a nominal P-value threshold. No SNPs survived the Bonferroni correction.

β value:

However, it's noteworthy that the *β* values of these significant SNPs exhibited a significant correlation (r=0.83; P-value<$1\text{x}10^{-10}$) between the two datasets. This observation underscores the importance of collecting genetic data within specific disease populations and throughout the entire lifespan (**Supplementary Figure 7** and **eFile 7**).

Supplementary note2: Exemplary genomic locus linked to GM, WM, and FC-BAG

The genomic locus (top lead SNP: rs534115641, **Fig. 2C**) linked to GM-BAG was mapped to multiple protein-encoding genes by position, eQTL, and chromatin interaction. The *NSF* gene, which encodes *N*-ethylmaleimide-sensitive fusion proteins, plays a key role in transferring membrane vesicles between cellular compartments. This gene has been linked to several conditions, including Parkinson's disease (PD)[5], epithelial ovarian cancer[6], cognitive traits[7], and fibromuscular dysplasia[8]. The *CRHR1* gene encodes a G protein-coupled receptor, which specifically binds to neuropeptides of the corticotropin-releasing hormone family. These neuropeptides are recognized as key regulators of the hypothalamic-pituitary-adrenal pathway. A prior GWAS[9] corroborated the association of this gene with the response to environmental stress, providing substantive support for the engagement of the hypothalamic-pituitary-adrenal axis, the central nervous system, and the endocrine system in regulating stress response[10]. We also identified a highly polygenic genomic locus (top lead SNP: rs564819152, **Fig. 2D**) for WM-BAG. This locus mapped to the *SKIDA1*, *CASC10*, *MLLT10*, and *DNAJC1* genes – all implicated in various types of cancer. In contrast, the FC-BAG locus was novel and did not map to any genes. All mapped genes for GM and WM-BAG are presented in **Supplementary Table 2**.

Supplementary figure 1: Split-sample genome-wide association results

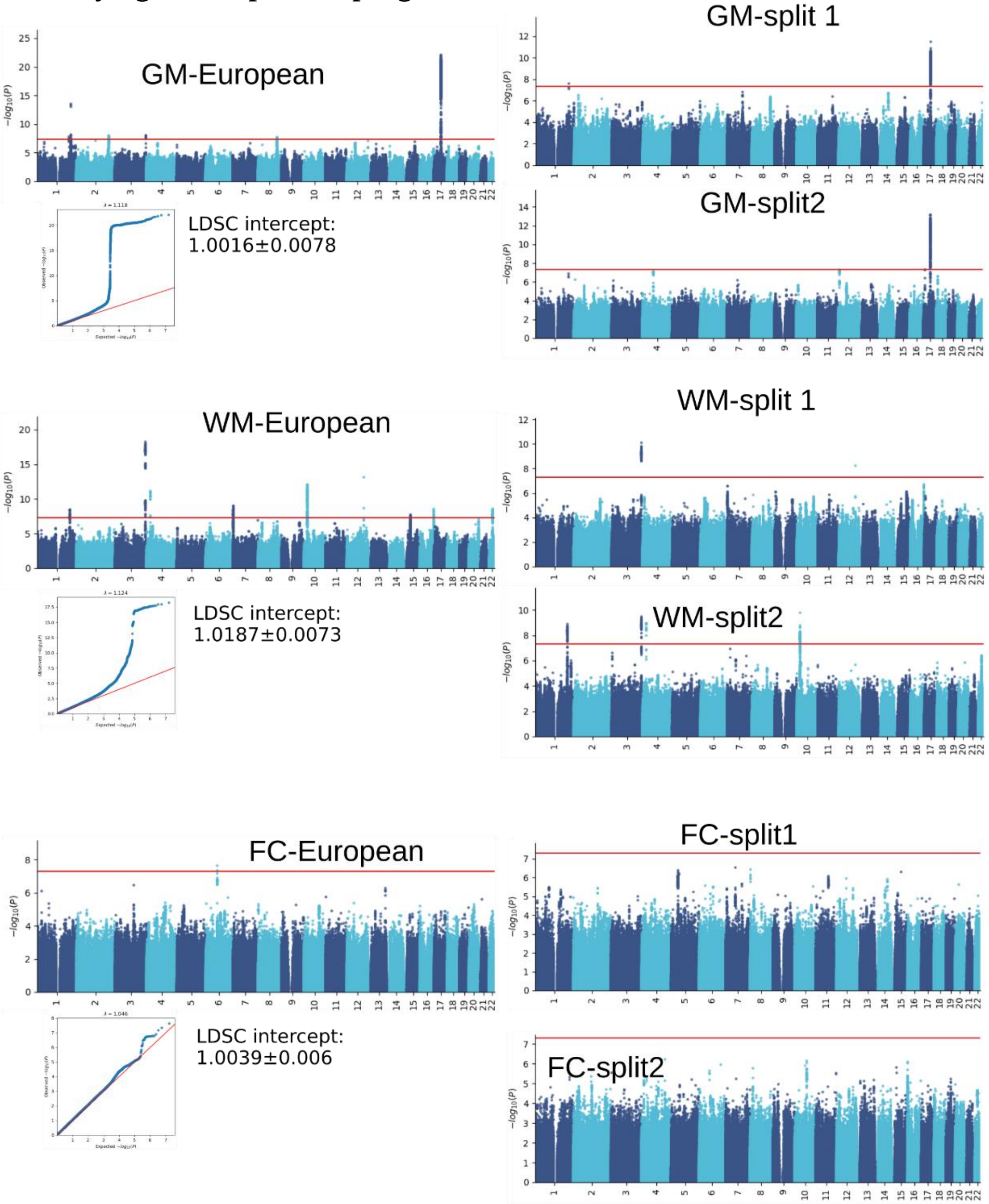

Genome-wide associations are presented for split-sample analyses (split1 vs. split2 vs. all). Genomic loci were identified using a genome-wide P-value threshold [$-\log_{10}$(P-value) > 7.30]. The sample sizes for GM-European, GM-split1, and GM-split2 are 31557, 15778, and 15778, respectively. The sample sizes for WM-European, WM-split1, and WM-split2 are 31674, 15837, and 15837, respectively. The sample sizes for FC-European, FC-split1, and FC-split2 are 32017, 16008, and 16008, respectively.

Supplementary figure 2: Sex-stratified genome-wide association results

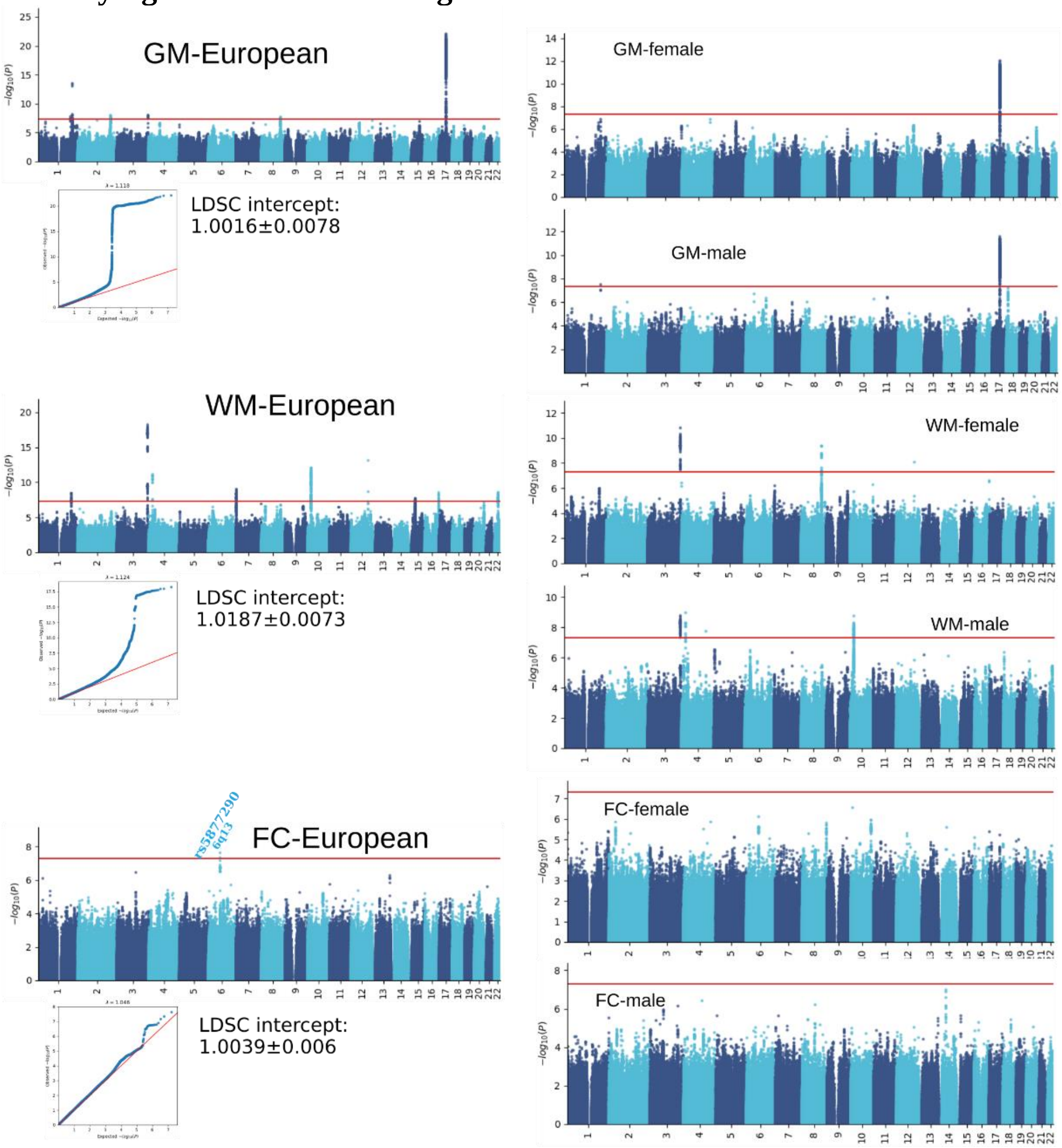

Genome-wide associations are presented for sex-stratified analyses (females vs. males). Genomic loci were identified using a genome-wide P-value threshold [$-\log_{10}$(P-value) > 7.30]. The sample sizes for GM-European, GM-female, and GM-male are 31557, 16558, and 14969, respectively. The sample sizes for WM-European, WM-female, and WM-male are 31674, 16693, and 14981, respectively. The sample sizes for FC-European, FC-female, and FC-male are 32017, 16890, and 15127, respectively.

Supplementary figure 3: Non-European genome-wide association results

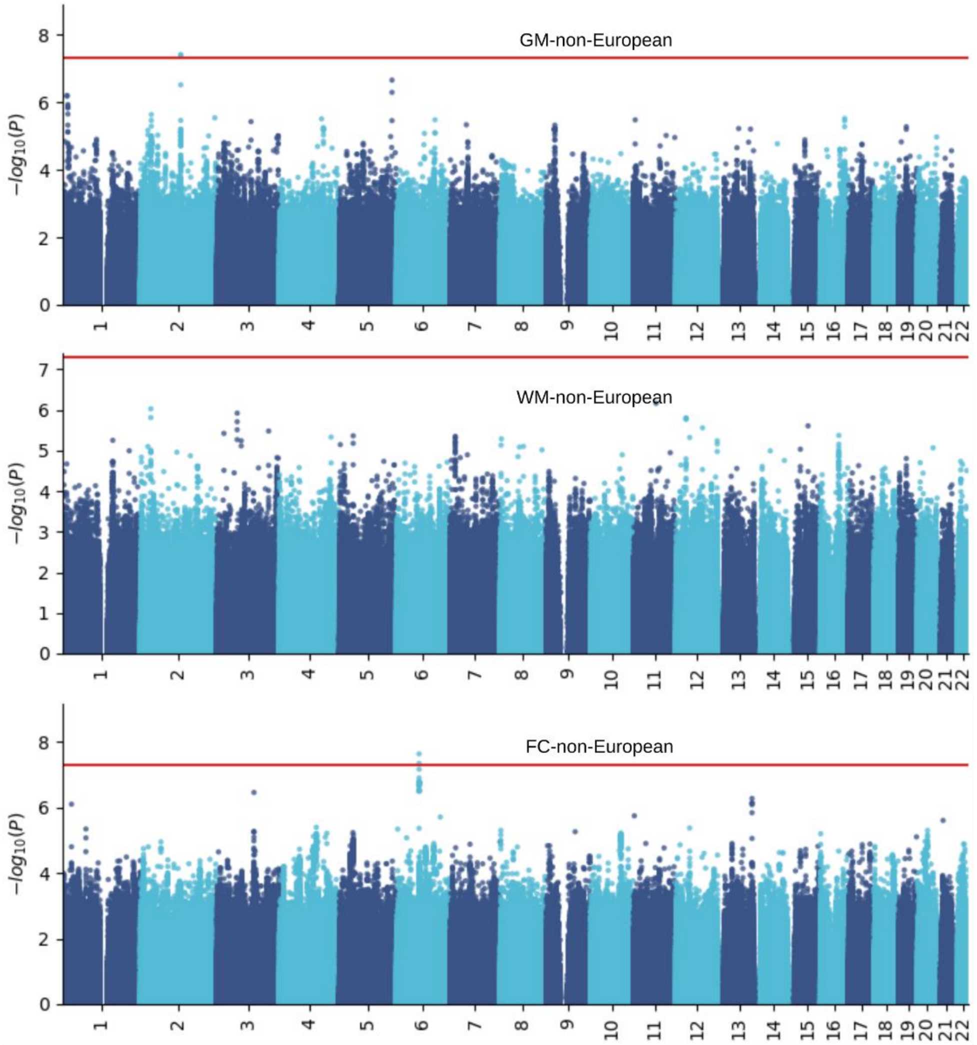

Genome-wide associations are presented for non-European populations in the UKBB study. Genomic loci associated were identified using a genome-wide P-value threshold [$-\log_{10}$(P-value) > 7.30]. The sample sizes for GM-non-European, WM-non-European, and FC-non-European are 4646, 5091, and 4728, respectively.

Supplementary figure 4: fastGWA for mixed linear models

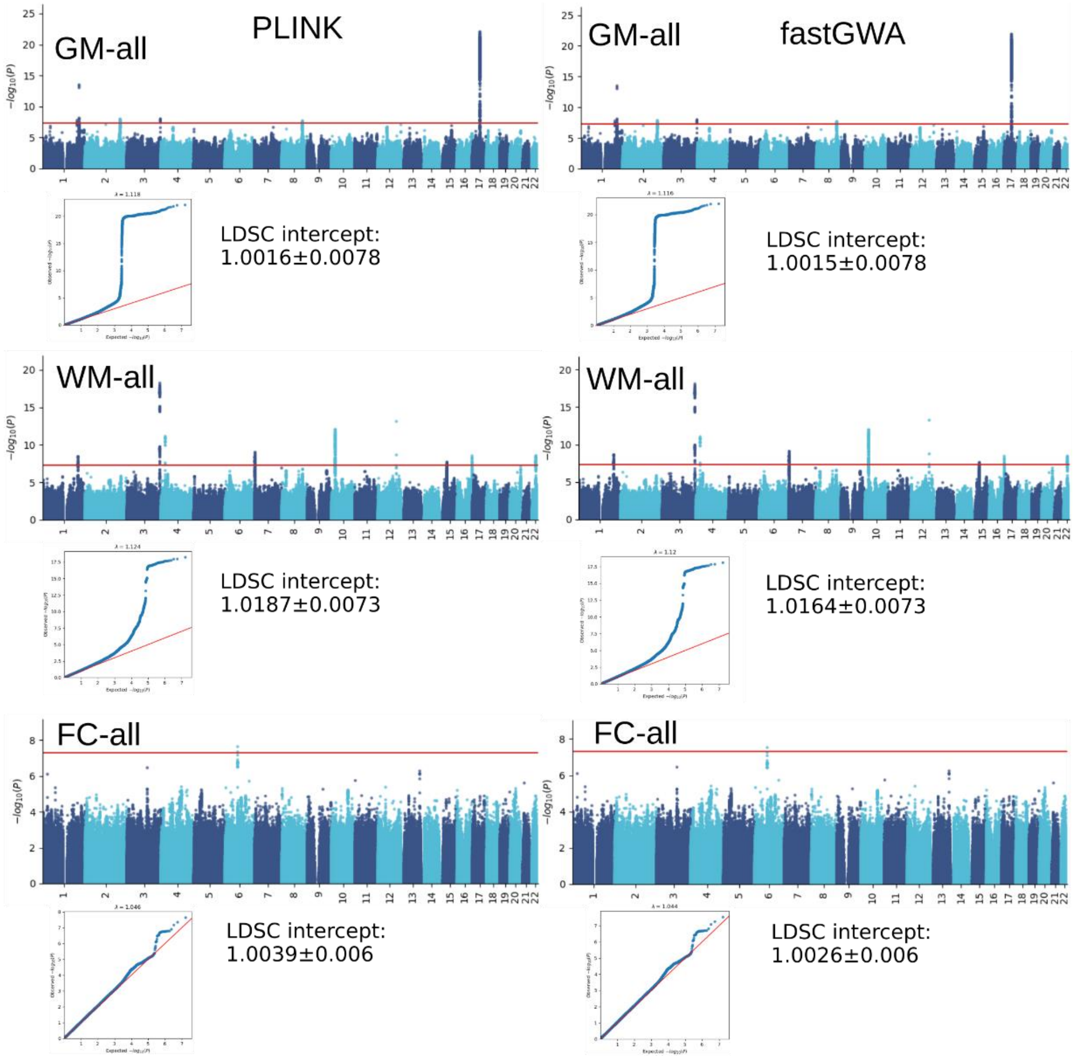

Genome-wide associations are presented for European populations in the UKBB study using fastGWA vs. PLINK. Genomic loci associated were identified using a genome-wide P-value threshold [$-\log_{10}$(P-value) > 7.30]. The sample sizes for GM-all, WM-all, and FC-all are 31557, 31674, and 32017 for PLINK, respectively. The sample sizes for GM-all, WM-all, and FC-all are 31557, 31674, and 32017 for fastGWA, respectively.

Supplementary figure 5: Machine learning-specific GWAS

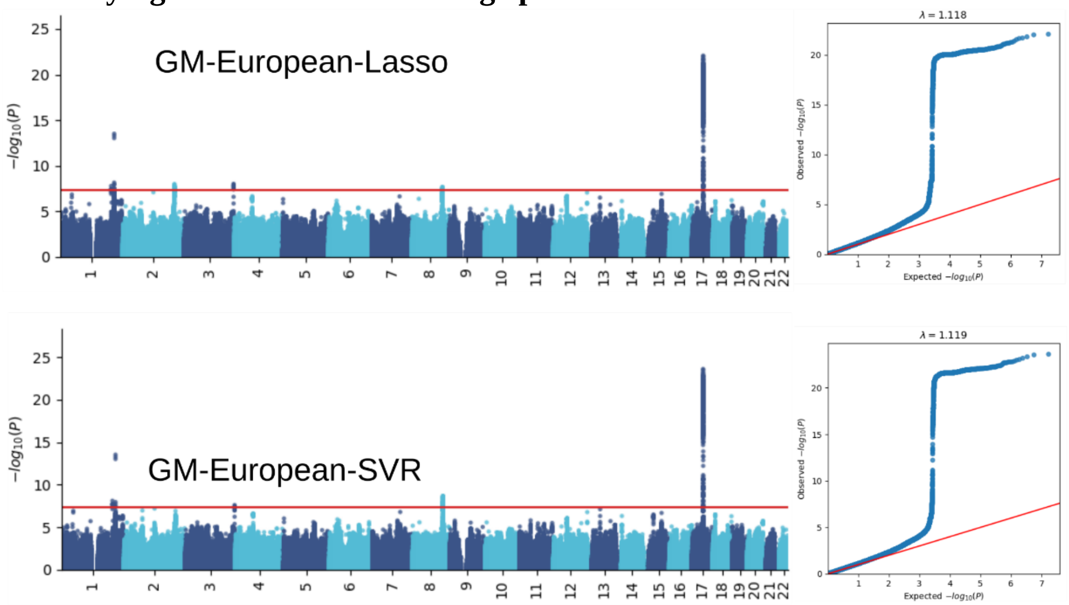

Genome-wide associations are presented for GM-BAG derived from Lasso regression (shown in the main text) and SVR, and CNN using voxel-wise images (GWAS summary statistics shared by a previous study which achieved an MAE ~ 2.5 years). Refer to **Fig. 1a** in the reference paper[2] for the Manhattan plot. Genomic loci associated were identified using a genome-wide P-value threshold [$-\log_{10}$(P-value) > 7.30]. The sample sizes for GM-European-Lasso and GM-European-SVR are 31557.

Supplementary figure 6: Feature type-specific GWAS

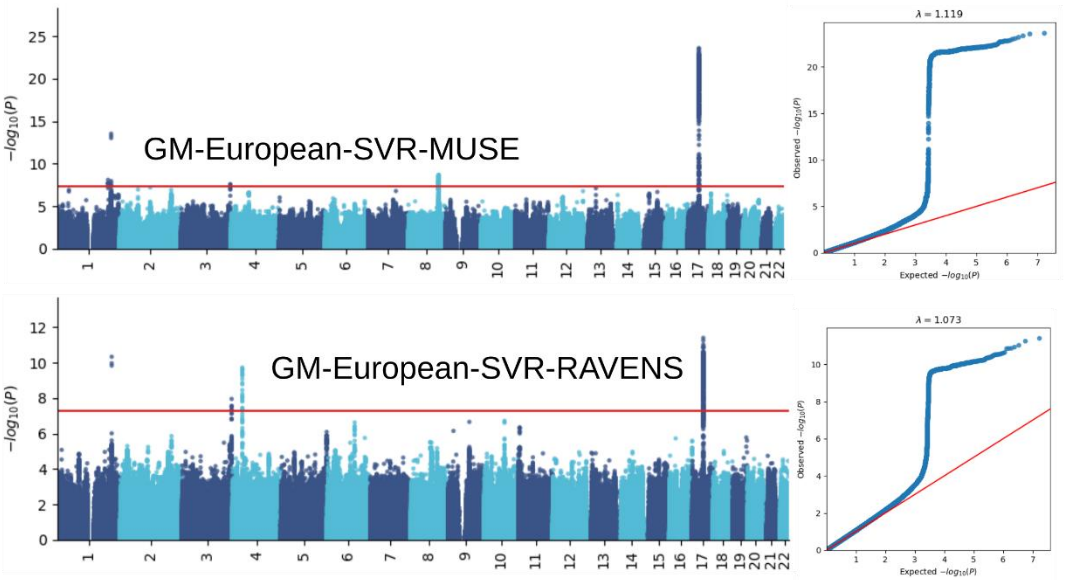

Genome-wide associations are presented for GM-BAG derived from MUSE ROIs (shown in the main text) and RAVENS voxel maps. Genomic loci associated were identified using a genome-wide P-value threshold [$-\log_{10}$(P-value) > 7.30]. The sample sizes for GM-European-SVR-MUSE and GM-European-SVR-RAVENS are 31557.

Supplementary figure 7: Independent WGS dataset for GM-BAG GWAS

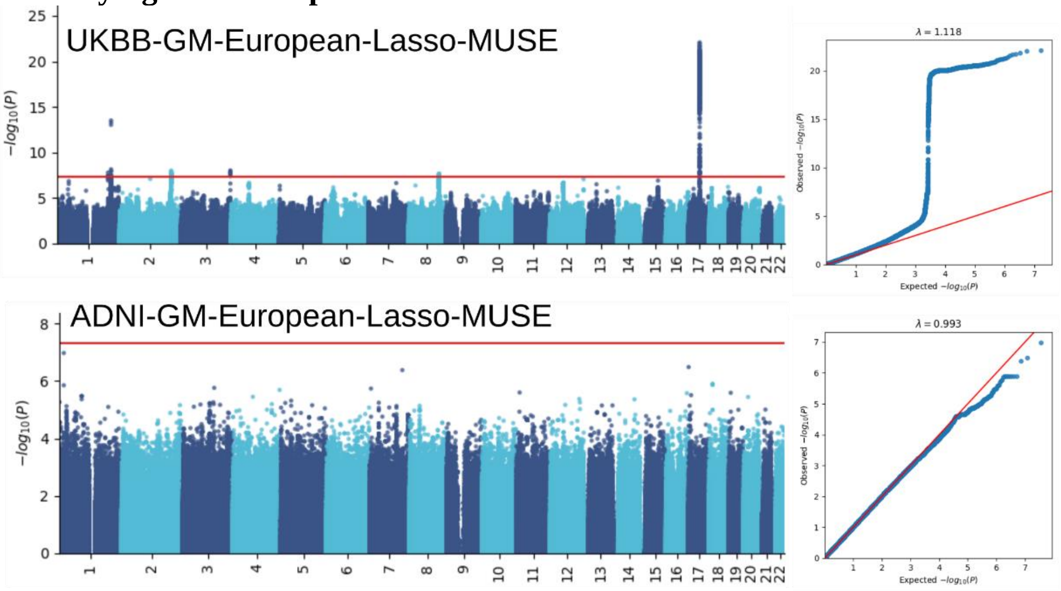

Genome-wide associations are presented for GM-BAG derived from MUSE ROIs (shown in the main text) using UKBB imputed genotyping data vs. ADNI WGS data. Genomic loci associated were identified using a genome-wide P-value threshold [$-\log_{10}$(P-value) > 7.30]. The genetic quality check steps for the ADNI GWAS are detailed elsewhwere[11]. The GM-BAG was generated by training a Lasso regression model from the ground up, utilizing ADNI healthy control participants, and achieving a similar Mean Absolute Error (MAE) of 4.24 years. However, when we directly applied the trained model to the ADNI population using UKBB data, we observed a higher MAE, ranging from 4.39 to 9.16 years. This discrepancy could potentially be attributed to the fact that ADNI participants tend to be older than those in the UKBB dataset. For ADNI WGS data, we first convert the VCF files into *plink* binary format. We excluded related individuals (up to 2nd-degree) using the KING software for family relationship inference.[10] Further QC steps are: excluding criteria were: i) individuals with more than 2% of missing genotypes; ii) variants with minor allele frequency (MAF) of less than 0.1%; iii) variants with larger than 5% missing genotyping rate; iv) variants that failed the Hardy-Weinberg test at $1x10^{-5}$. We then removed duplicated variants from all 22 autosomal chromosomes. We also excluded individuals for whom either imaging or genetic data were not available. To adjust for population stratification,[11] we derived the first 40 genetic principal components (PC) using the SmartPCA software[12]. The sample sizes for UKBB-GM-European-Lasso-MUSE and ADNI-GM-European-Lasso-MUSE are 31557 and 1104, respectively.

Supplementary figure 8: Genetic correlation (g_c) between the GM, WM, and FC-BAG using the LDSC software in the split-sample analyses

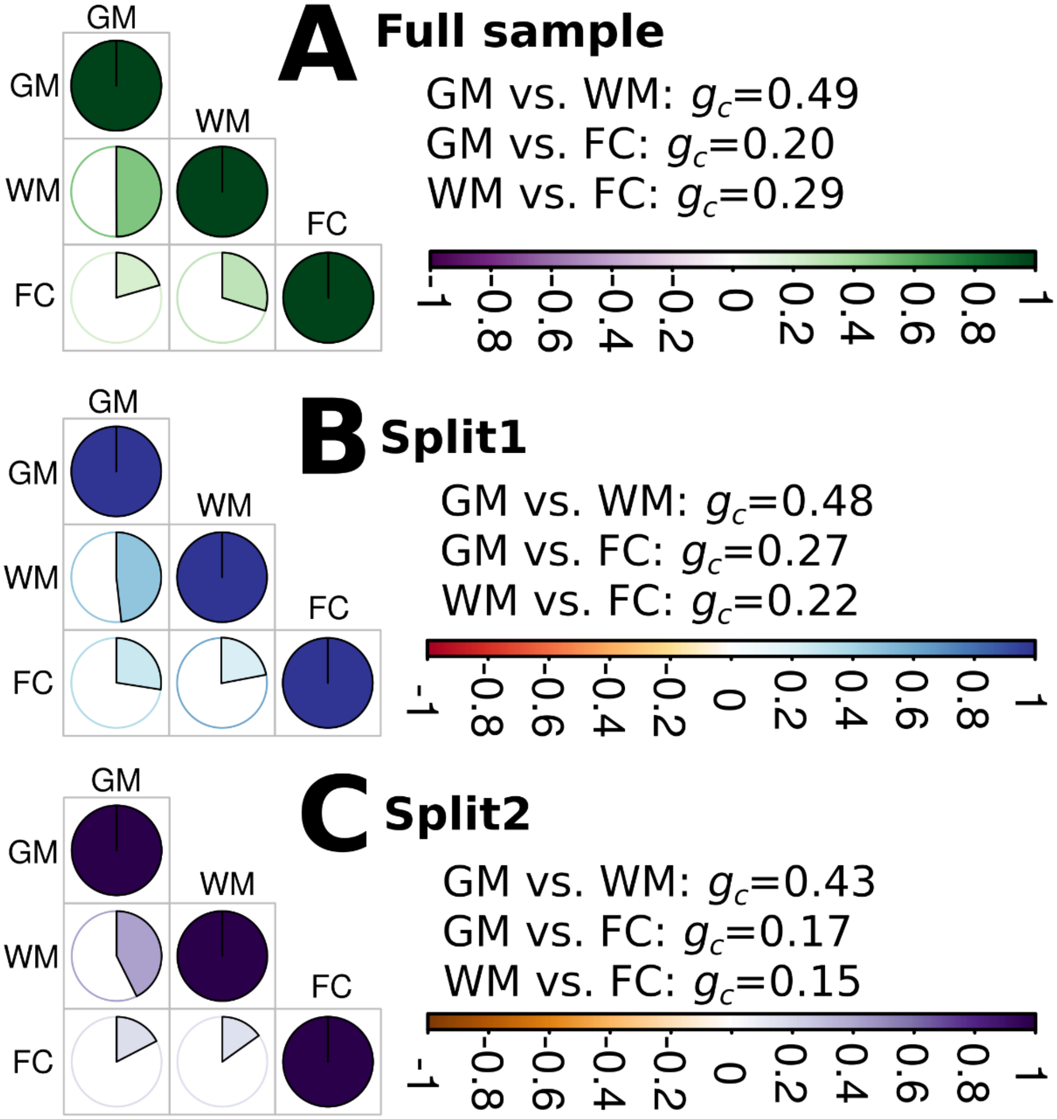

A) Genetic correlation using the full samples. **B**) Genetic correlation using the split1 sample. **C**) Genetic correlation using the split2 sample.

Supplementary figure 9: Incremental R2 of the PRS derived by the PLINK C+T approach

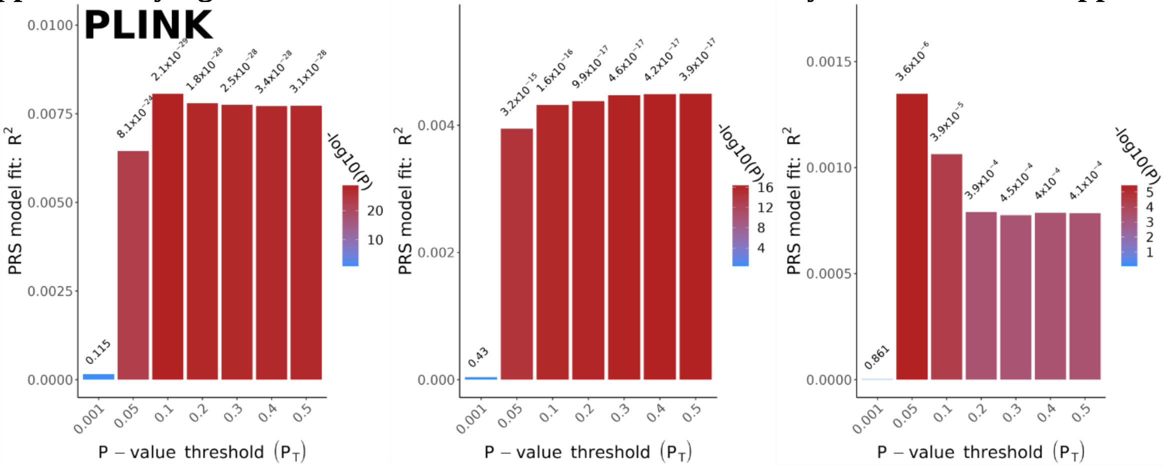

Incremental R^2 of the PRS derived by the PLINK C+T approach to predict the GM, WM, and FC-BAG in the target/test data (i.e., the split2 GWAS population in the split-sample analyses). The *y*-axis indicates the proportions of phenotypic variation (GM, WM, and FC-BAG) that the PRS can significantly and additionally explain. The *x*-axis lists the seven P-value thresholds considered.

Supplementary figure 10: Results for the inverse Mendelian randomization for the seven clinical traits

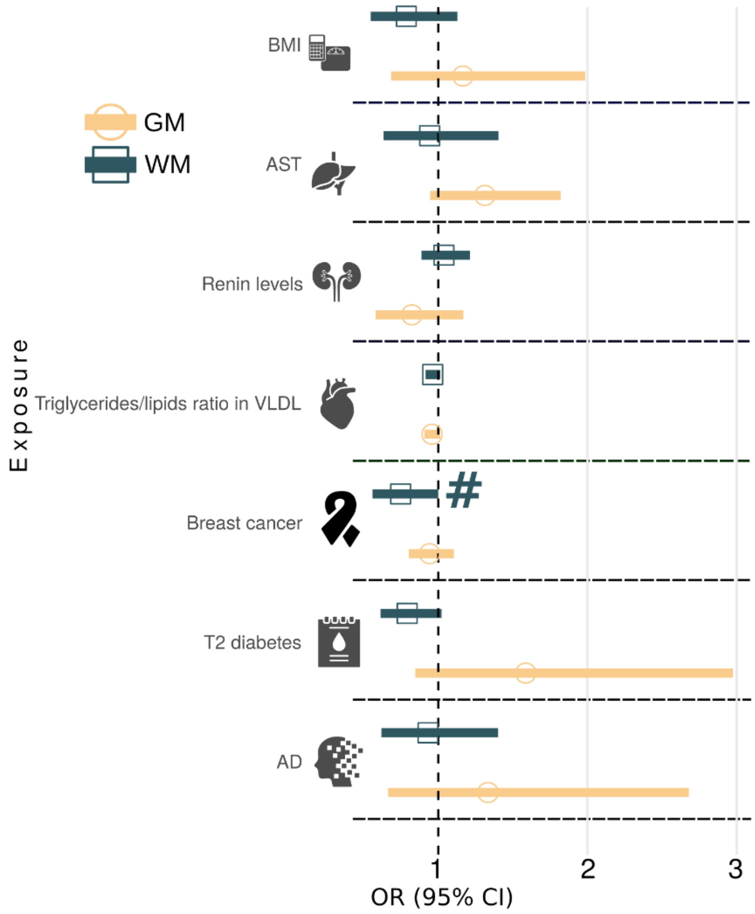

The inverse causal inference was performed using a two-sample Mendelian Randomization approach for seven selected clinical traits as outcome variables and GM, WM, and FC-BAG as exposure variables. Shapes (circle and rectangle) represent the Odds Ratio (OR), and its 95% confidence interval (CI) is also presented. The symbol # indicates that the tests pass the nominal P-value threshold (two-sided) of 0.05 but do not survive the FDR correction. Abbreviation: AD: Alzheimer's Disease; AST: Aspartate Aminotransferase; BMI: Body Mass Index; VLDL: Very Low-Density Lipoprotein. The exact statistics (e.g., P-value) are shown in Supplementary Data 12.

Supplementary figure 11: Sensitivity check for all other significant exposure variables in the forward MR analyses for 1) breast cancer on GM-BAG, 2) diabetes on GM-BAG, and 3) AD on WM-BAG.

1) Sensitivity checks of causal effects of breast cancer on GM-BAG. **A**) Scatter plot indicates one potential outlier. **B**) Funnel plot shows no obvious asymmetry and points out two outliers. **C**) Single-SNP MR results. **D**) Leave-one-out analyses. Each dot represents the mean value of the estimated parameters, and the error bar displays its 95% confidence interval (**C, D**) or standard errors of the parameters (**A**).

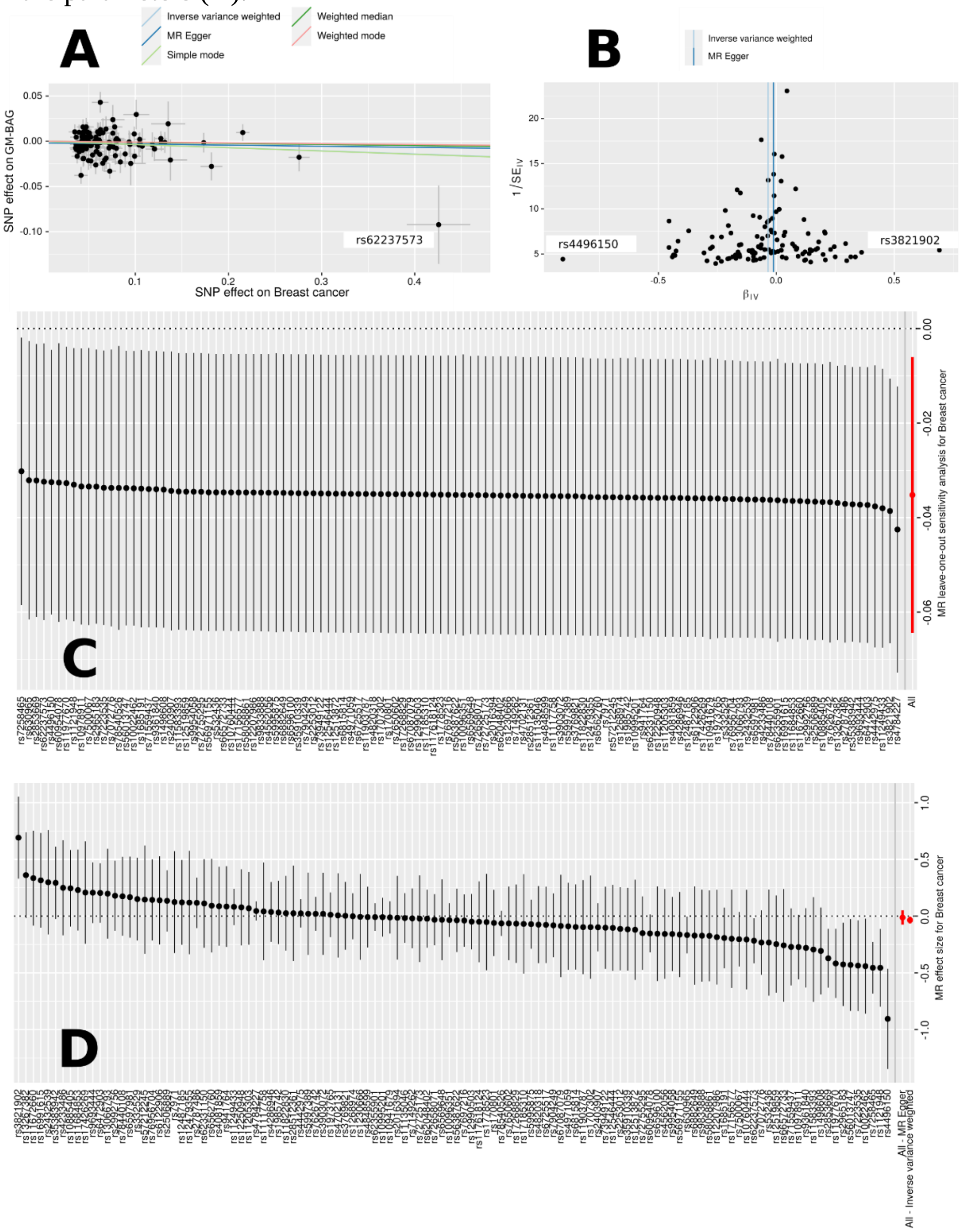

2) Sensitivity checks of causal effects of type 2 diabetes on GM-BAG. **A**) Scatter plot for the heterogeneity of the causal effects. **B**) Funnel plot shows the asymmetry of the causal effects. **C**) Single-SNP MR results. **D**) Leave-one-out analyses. Each dot represents the mean value of the estimated parameters, and the error bar displays its 95% confidence interval (**C, D**) or standard errors of the parameters (**A**).

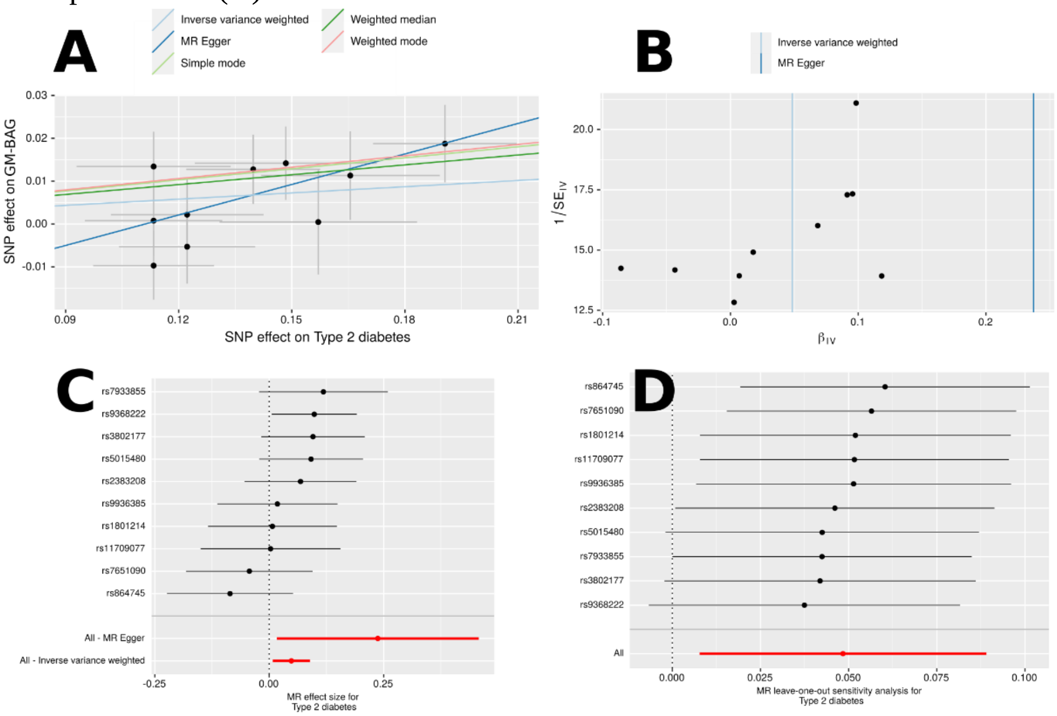

3) Sensitivity checks of causal effects of AD on WM-BAG. **A**) Scatter plot for the heterogeneity of the causal effects. **B**) Funnel plot shows no apparent asymmetry of the causal effects. **C**) Single-SNP MR results. **D**) Leave-one-out analyses. Each dot represents the mean value of the estimated parameters, and the error bar displays its 95% confidence interval (**C, D**) or standard errors of the parameters (**A**).

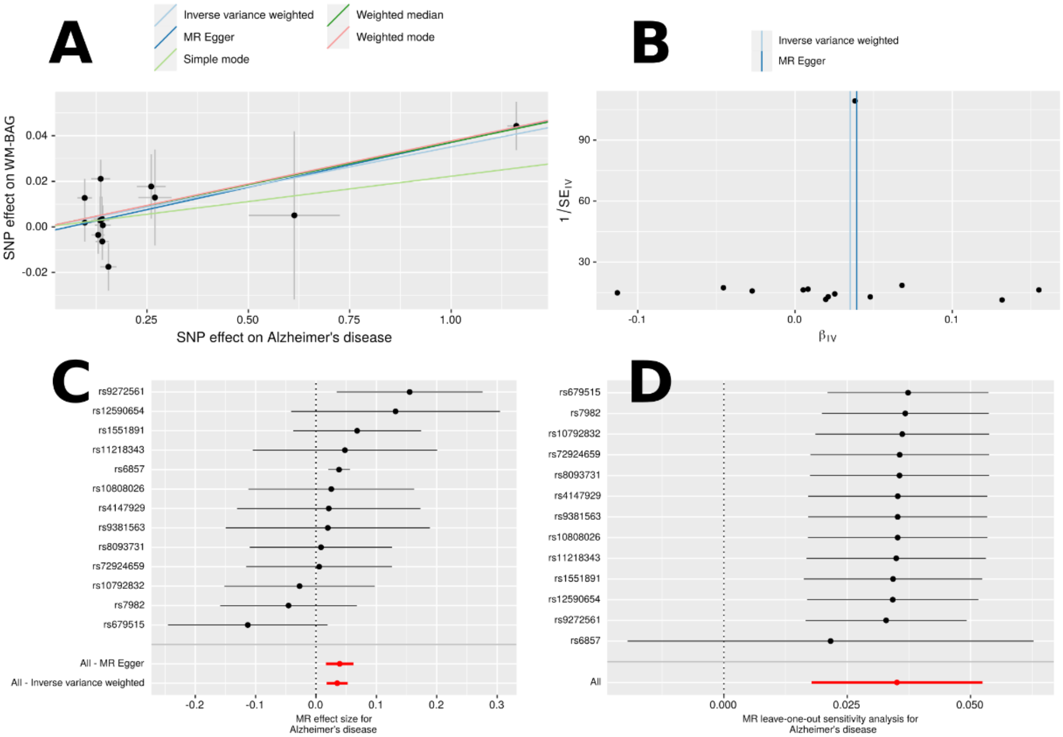

Supplementary figure 12: RNA expression overview of the *DNAJC1* gene in various cancer types.

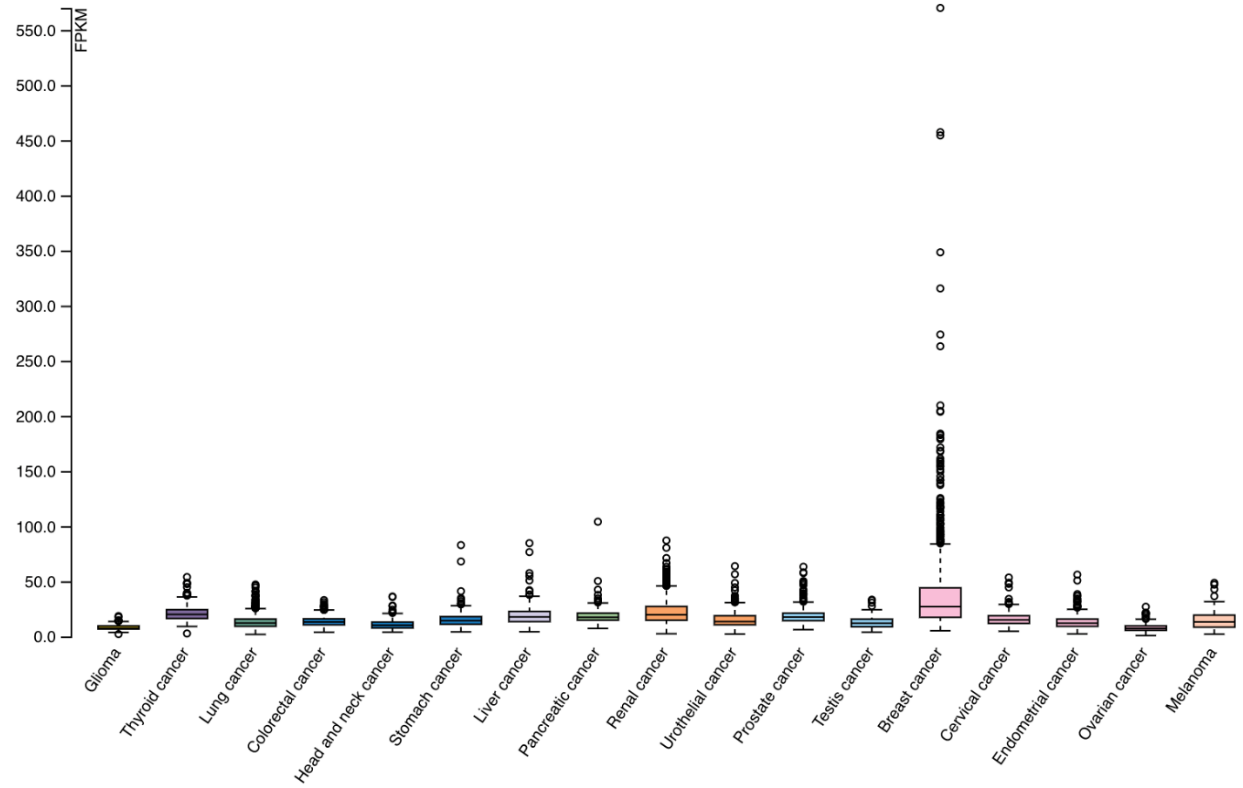

RNA expression overview shows RNA-seq data from The Cancer Genome Atlas (TCGA) project. FPKM represents fragments per kilobase of transcript per million mapped reads. The estimated gene expression values of the *DNAGC1* gene are displayed for 17 types of cancer/tumors.

Supplementary figure 13: The five-layer neural network used for age prediction and its performance using WM-IDP

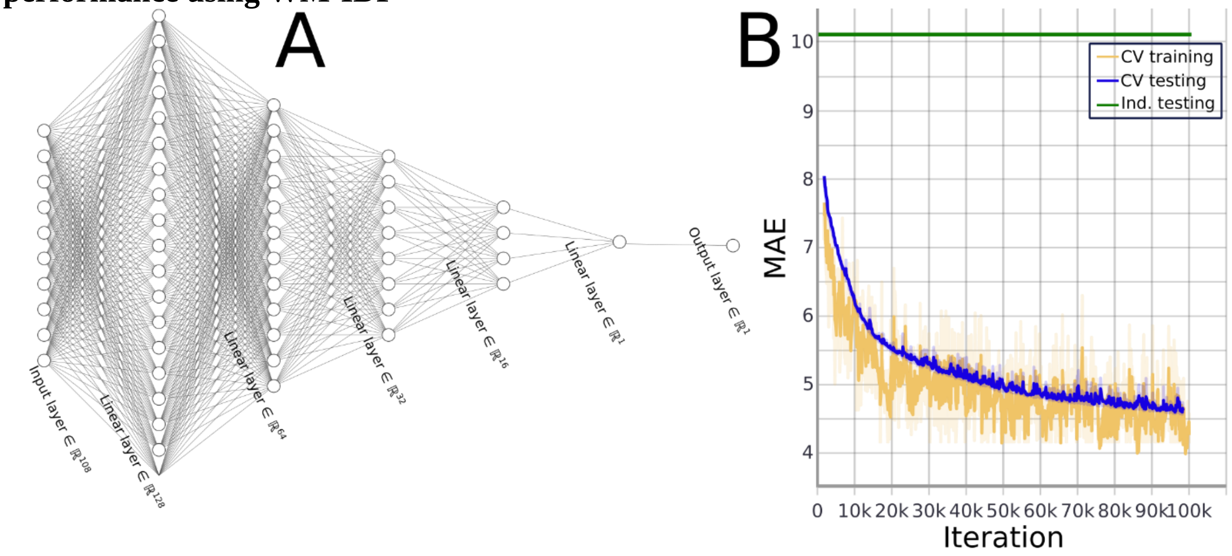

A) We illustrate the neural network architecture utilized in this study to predict brain age using GM, WM, and FC-IDP. The dimensionality of neurons in each linear layer is indicated in the diagram. **B**) We present the results of the cross-validation (CV) training, CV testing, and independent testing loss for the WM-IDP using FA, MD, ODI, and NDI from the TBSS-based approach. Notably, the network overfits the 108 WM-IDP since the number of network parameters (38,364) is significantly greater than the number of features (108). In addition, features from FA, MD, ODI, and NDI are highly correlated.

Supplementary table 1: Brain age prediction performance using GM, WM, and FC-IDP. We reported each machine learning model's mean absolute errors (MAE, year) and Pearson's correlation coefficient (r). The cross-validated (CV Test) and independent (Ind. Test) testing results were shown. Table **A** shows the results from the CV Test and the independent (Ind.) Test performance. Table **B** contains the results of the sex-stratified experiments.

A: **Brain age prediction results from the CV and independent test dataset**. The bolded text represents the lowest MAE for IDPs from each MRI modality. For WM-IDP, we fit the models with different combinations of features: i) 108 weighted mean TBSS WM-IDP from FA, MD, OD, and NDI; ii) 192 skeleton mean values of WM-IDP from FA, MD, OD, and NDI; iii) 48 FA WM-IDP.

IDP		Dataset	Linear SVR		Lasso regression		MLP		NN	
			MAE	*r*	MAE	*r*	MAE	*r*	MAE	*r*
GM-IDP		CV Test	4.86±0.11	0.77	4.92±0.11	0.77	4.88±0.13	0.77	4.42±0.11	0.79
		Ind. Test	4.43	0.66	**4.39**	**0.66**	5.35	0.64	4.83	0.65
WM-IDP	108 TBSS	CV Test	4.88±0.11	0.77	4.94±0.11	0.78	5.29±0.13	0.75	4.54±0.11	0.79
		Ind. Test	5.27	0.53	6.29	0.53	7.41	0.59	10.12	0.31
	192 FA/MD/ODI/NDI	CV Test	4.07±0.10	0.84	4.142±0.11	0.84	4.34±0.12	0.83	3.50±0.10	0.87
		Ind. Test	21.90	0.71	21.66	0.73	6.12	0.71	15.77	0.30
	48 FA	CV Test	5.12±0.12	0.75	5.15±0.11	0.75	5.32±0.13	0.73	6.85±0.15	0.56
		Ind. Test	5.02	0.65	**4.92**	**0.65**	7.95	0.60	6.84	0.42
FC-IDP		CV Test	6.28±0.16	0.58	6.51±0.16	0.59	6.07±0.17	0.66	5.88±0.15	0.63
		Ind. Test	5.97	0.43	**5.48**	**0.44**	6.02	0.46	6.05	0.43

B: Brain age prediction results from the sex-stratified experiments.

IDP	Gender	Set	Linear SVR		Lasso regression		MLP		NN	
			MAE	*r*	MAE	*r*	MAE	*r*	MAE	*r*
GM-IDP	Female	CV Test	4.77±0.18	0.77	4.89±0.17	0.77	4.93±0.23	0.76	4.25±0.16	0.80
		Ind. Test	4.46	0.64	4.44	0.64	6.94	0.60	5.02	0.62
	Male	CV Test	4.69±0.16	0.78	4.77±0.16	0.79	4.70±0.22	0.79	4.08±0.17	0.82
		Ind. Test	4.58	0.65	4.49	0.66	5.03	0.65	4.85	0.64
WM-IDP	Female	CV Test	4.81±0.16	0.78	4.77±0.17	0.79	4.73±0.17	0.78	5.61±0.17	0.66
		Ind. Test	25.97	0.61	26.39	0.62	31.68	0.56	19.21	0.55
	Male	CV Test	4.83±0.21	0.77	4.86±0.25	0.79	5.24±0.26	0.75	5.80±0.19	0.68
		Ind. Test	7.88	0.63	5.67	0.62	10.24	0.62	14.96	0.56
FC-IDP	Female	CV Test	5.85±0.21	0.62	6.01±0.20	0.64	6.41±0.23	0.61	5.07±0.20	0.69
		Ind. Test	5.93	0.42	5.58	0.42	5.36	0.42	6.28	0.41
	Male	CV Test	6.03±0.23	0.60	6.32±0.22	0.62	6.83±0.23	0.59	6.11±0.22	0.62
		Ind. Test	6.01	0.40	5.64	0.41	6.79	0.42	5.78	0.41

Supplementary table 2: Identified genomic loci and mapped genes.

Two-siede P-values were derived from our linear regression GWAS.

GM-BAG:

Locus	Top lead SNP	P-value	Chromosome	Mapped genes
1	rs61732315	$1.63x10^{-8}$	1	*MYOG, PPFIA4, ADORA1*
2	rs1452628	$3.04x10^{-14}$	1	*KCNK2, KCTD3*
3	rs186399184	$1.05x10^{-8}$	2	*CYP20A1, CARF, FAM117B, WDR12, ABI2, NBEAL1, ICA1L*
4	rs10933668	$9.41x10^{-9}$	3	NA
5	rs34051980	$2.02x10^{-8}$	8	*TNFRSF11B, COLEC10*
6	rs534115641	$8.44x10^{-23}$	17	*NSF, WNT3, KANSL1, CRHR1, NMT1, ARHGAP27, LRRC37A, EFCAB13, C17orf104, FMNL1, SPPL2C, ARL17A, MAPT, PLEKHM1, ARL17B, LRRC37A2, STH*

WM-BAG:

Locus	Top lead SNP	P-value	Chromosome	Mapped genes
1	rs11118475	$3.69x10^{-09}$	1	*CD46, CR1L*
2	rs61067594	$6.01x10^{-19}$	3	*GMNC*
3	rs967140	$7.26\ x10^{-12}$	4	*PPARGC1A*
4	rs2533872	$9.95\ x10^{-10}$	7	*GNA12, AMZ1*
5	rs564819152	$9.39\ x10^{-13}$	10	*SPAG6, MLLT10, DNAJC1, COMMD3, BMI1, SKIDA1, CASC10, COMMD3-BMI1*
6	rs12146713	$7.67\ x10^{-14}$	12	*NUAK1*
7	rs654276	$1.96\ x10^{-08}$	15	*TP53BP1, WDR76, ELL3, TUBGCP4, MFAP1, SERF2, ZSCAN29, TGM7, CASC4, CATSPER2, MAP1A, PDIA3, PPIP5K1, ADAL, LCMT2, FRMD5, SERINC4, CKMT1A, CKMT1B, HYPK, STRC, RP11-296A16.1, AC018512.1*
8	rs4843550	$2.84\ x10^{-09}$	16	*C16orf95*
9	rs1894525	$2.71x10^{-09}$	22	*NOL12, TRIOBP, GCAT, ANKRD54, EIF3L, MICALL1, PICK1, GALR3, H1F0*

FC-BAG:

Locus	Top lead SNP	P-value	Chromosome	Mapped genes
1	rs5877290	$2.31x10^{-8}$	6	NA

Supplementary table 3: Selected clinical traits for genetic correlation analyses. We selected the candidate studies from the GWAS Catalog for specific traits, including neurodegenerative diseases, psychiatric disorders, education, and intelligence. The inclusion criteria are i) GWAS summary statistics are publicly available; ii) the study population is European ancestry in the majority; iii) the heritability estimates (h^2) via LDSC are not spuriously low (h^2>0.05). This resulted in six clinical traits. We present the clinical trait, the dataset used, the URL link, the Pubmed ID, and the sample size. Abbreviations: PGC: psychiatric genomics consortium; ADHD: attention deficit hyperactivity disorder; ASD: autism spectrum disorder; MDD: major depressive disorder; OCD: obsessive-compulsive disorder; SCZ: schizophrenia; BPD: bipolar disorder; SSGAC: Social Science Genetic Association Consortium; UKBB: UK Biobank.

Trait	Dataset	URL	PubMed ID/GWAS Catalog ID	Sample size
AD	Meta	http://ftp.ebi.ac.uk/pub/databases/gwas	30820047	63,926
AD subtypes	UKBB	https://labs.loni.usc.edu/medicine/organ_systems/brain	NA	33,540
ADHD	PGC	https://figshare.com/articles/dataset/adhd2019/14671965	30478444	53,293
ASD	PGC	https://figshare.com/articles/dataset/asd2019/14671989	30804558	46,350
ASD subtypes	UKBB	https://labs.loni.usc.edu/medicine/organ_systems/brain	37017948	14,786
BPD	PGC	https://figshare.com/articles/dataset/bip2019/14671998	31043756	51,710
MDD	Meta	https://figshare.com/articles/dataset/mdd2013/14672082	22472876	18,759
Education	SSGAC	http://ftp.ebi.ac.uk/pub/databases/gwas	23722424	126,559
Intelligence	CTGlab	http://ftp.ebi.ac.uk/pub/databases/gwas	28530673	78,308
SCZ	PGC	https://figshare.com/articles/dataset/scz2013sweden/14672154	23974872	11,244
SCZ subtypes	UKBB	https://www.cbica.upenn.edu/bridgeport	32103250	14,786
OCD	Meta	https://figshare.com/articles/dataset/ocd2018/14672103	28761083	9,725

Supplementary table 4: Selected exposure variables for the forward Mendelian randomization. We present here the traits, searching keywords, PubMed ID of the study, and the IEU ID. MR mimics randomized clinical trials using genetic variants (SNP) randomly allocated at conception as instrumental variables (IV) to estimate the causal effect of an exposure (e.g., alcohol consumption) on an outcome (e.g., GM-BAG). In essence, MR is less prone to confounding and reverse causation bias. Genetic variants, however, must be associated with the exposure variable (relevance assumption), not associated with the outcome biased by confounders (exchangeability assumption), and only associated with the outcome through the exposure (exclusivity assumption). In particular, we automatically queried these traits in the IEU GWAS database[12] – curated GWAS summary statistics for MR – to extract the IVs from i) European ancestry, ii) non-UKBB studies (our GWAS were derived from UKBB data), iii) and large sample sizes. Another rationale for performing this hypothesis-driven MR analysis was the extensive coverage of UK Biobank (UKBB) in the IEU GWAS database, necessitating the exclusion of UKBB-based GWAS from our analysis to mitigate potential biases associated with sample overlap. AD: Alzheimer's disease.

Trait	Searching keyword	PubMed ID	IEU ID	N
AD	Alzheimer	24162737	ebi-a-GCST002245	17008 AD and 37154 controls
Breast cancer	cancer	29059683	ieu-a-1126	122,977 cases and 105,974 controls
Type 2 diabetes	diabetes	22885922	ieu-a-26	34,840 cases and 114,981 controls
Renin level	Renin	33067605	ebi-a-GCST90012038	30,931
Triglyceride-to-lipid ratio	Triglyceride	32114887	met-d-XL_VLDL_TG_pct	16,126
AST	Aspartate aminotransferase	29875488	prot-a-1241	3301
BMI	Body mass index	23563607	ieu-a-85	263,407

Supplementary table 5: Study characteristics.
The current table presents participants of all ancestries for the age prediction task. We constrained participants with only European ancestry for downstream genetic analyses. * For age and sex, we reported statistics for the overlapping population of the three modalities: 35,261 participants for the entire population, 4000 participants for the training/validation/test dataset, and 31,261 participants for the independent test dataset. We also showed the number of participants for the GM, WM, and FC-BAG GWAS. In total, our analyses included 42,089 unique participants who had at least one image scan. Abbreviation: dMRI: diffusion MRI; rsfMRI: resting-state functional MRI; T1w MRI: T1-weighted MRI.

Population (overlap)	T1w MRI	dMRI	rsfMRI	Age (year)*	Sex /female*
Total (35,261)	36,304	39,661	36,858	63.64 (45.00, 81.00)	18,700/53%
Training/validation/test (4000)	4000	4000	4000	63.47 (46.00, 81.00)	2000/50%
Independent test (31,261)	32,304	35,661	32,858	63.66 (45.00, 81.00)	16,700/53%
GWAS	31,557	31,749	32,017	NA	NA

References

1. Jiang, L. *et al.* A resource-efficient tool for mixed model association analysis of large-scale data. *Nat Genet* **51**, 1749–1755 (2019).
2. Leonardsen, E. H. *et al.* Genetic architecture of brain age and its causal relations with brain and mental disorders. *Mol Psychiatry* 1–10 (2023) doi:10.1038/s41380-023-02087-y.
3. Doshi, J. *et al.* MUSE: MUlti-atlas region Segmentation utilizing Ensembles of registration algorithms and parameters, and locally optimal atlas selection. *Neuroimage* **127**, 186–195 (2016).
4. Davatzikos, C., Genc, A., Xu, D. & Resnick, S. M. Voxel-Based Morphometry Using the RAVENS Maps: Methods and Validation Using Simulated Longitudinal Atrophy. *NeuroImage* **14**, 1361–1369 (2001).
5. Hamza, T. H. *et al.* Common genetic variation in the HLA region is associated with late-onset sporadic Parkinson's disease. *Nat Genet* **42**, 781–785 (2010).
6. Kuchenbaecker, K. B. *et al.* Identification of six new susceptibility loci for invasive epithelial ovarian cancer. *Nat Genet* **47**, 164–171 (2015).
7. de la Fuente, J., Davies, G., Grotzinger, A. D., Tucker-Drob, E. M. & Deary, I. J. A general dimension of genetic sharing across diverse cognitive traits inferred from molecular data. *Nat Hum Behav* **5**, 49–58 (2021).
8. Georges, A. *et al.* Genetic investigation of fibromuscular dysplasia identifies risk loci and shared genetics with common cardiovascular diseases. *Nat Commun* **12**, 6031 (2021).
9. Nagel, M., Speed, D., van der Sluis, S. & Østergaard, S. D. Genome-wide association study of the sensitivity to environmental stress and adversity neuroticism cluster. *Acta Psychiatrica Scandinavica* **141**, 476–478 (2020).

10. Russell, G. & Lightman, S. The human stress response. *Nat Rev Endocrinol* **15**, 525–534 (2019).

11. Wen, J. *et al.* Genetic, clinical underpinnings of subtle early brain change along Alzheimer's dimensions. 2022.09.16.508329 Preprint at https://doi.org/10.1101/2022.09.16.508329 (2022).

12. Elsworth, B. *et al.* The MRC IEU OpenGWAS data infrastructure. 2020.08.10.244293 Preprint at https://doi.org/10.1101/2020.08.10.244293 (2020).
