# Supplementary material for: The genetic architecture of multimodal human brain age
Source: Nat Commun. 2024 Mar 23;15:2604. doi: 10.1038/s41467-024-46796-6 (PMC10960798; doi:10.1038/s41467-024-46796-6)
Supplement: Supplementary file 3 — Description of Additional Supplementary Files [file 41467_2024_46796_MOESM3_ESM.pdf]

Description of Additional Supplementary Files

File Name: Supplementary Data 1
Description: The table contains the genome-wide associations for the split1 GWAS (Discovery) and split2 GWAS (Replication) in the split-sample GWAS. We present the results for SNPs passing the genome-wide P-value threshold (5x10-8) in the Discovery.

File Name: Supplementary Data 2
Description: The table contains the genome-wide associations between the female-GWAS (Discovery) and male-GWAS (Replication) in sex-stratified GWAS. We present the results for SNPs passing the genome-wide P-value threshold (5x10-8) in the Discovery.

File Name: Supplementary Data 3
Description: The table contains the genome-wide associations between the European GWAS (Discovery) and non-European GWAS (Replication) in ancestry-specific GWAS. We present the results for SNPs passing the genome-wide P-value threshold (5x10-8) in the Discovery.

File Name: Supplementary Data 4
Description: The table contains the genome-wide associations between the PLINK GWAS (Discovery) and fastGWA (Replication) for GWAS method-specific GWAS. We present the results for SNPs passing the genome-wide P-value threshold (5x10-8) in the Discovery.

File Name: Supplementary Data 5
Description: The table contains the genome-wide associations for the GM-BAG GWAS using Lasso regression (Discovery) and SVR (Replication) on the left table, and Lasso regression (Discovery) vs. CNN (replication) on the right table, for ML method-specific GWAS. We present the results for SNPs passing the genome-wide P-value threshold (5x10-8) in the Discovery.

File Name: Supplementary Data 6
Description: The table contains the genome-wide associations for the GM-BAG GWAS using MUSE ROI (Discovery) and voxel-wise RAVENS maps (Replication) for imaging feature-specific GWAS. We present the results for SNPs passing the genome-wide P-value threshold (5x10-8) in the Discovery.

File Name: Supplementary Data 7
Description: The table contains the genome-wide associations for the GM-BAG GWAS using UKBB imputed data (Discovery) and ADNI WGS data (Replication) for dataset-specific GWAS. We present the results for SNPs passing the genome-wide P-value threshold (5x10-8) in the Discovery.

File Name: Supplementary Data 8
Description: The table contains the phenome-wide association query in GWAS Catalog using the candidate, independent (Ind.) Significant (Sig.) SNPs within each locus linked to GM, WM, and FC-BAG in our GWAS. This phenome-wide query was performed using the functionality in FUMA.

File Name: Supplementary Data 9
Description: The table contains the results of the gene-drug-disease network. We curated data from the Drug Bank database (v.5.1.9) and the Therapeutic Target Database (updated by September 29th, 2021) to construct a gene-drug-disease network.

File Name: Supplementary Data 10
Description: Results for genetic correlation estimates for the 16 clinical traits. We reported the genetic correlation (r_g) estimates, their standard errors, and the Z and P-values (two-sided). The details of the selected traits are presented in Supplementary Table 3. We also presented the h^2 estimates using the LDSC software. Abbreviation: BAG: brain age gap.

File Name: Supplementary Data 11
Description: Results for partitioned heritability estimates for the 53 functional categories (A) and cell type-specific analysis (B). We reported the partitioned heritability estimates, the standard errors, and the Z and P-values (one-sided). In total, there are 52 categories presented here, as we did not show the results for all available SNPs (the baseline model) for Table A.

File Name: Supplementary Data 12
Description: The table contains the results for the bidirectional Mendelian randomization: forward and the inverse MR by switching the exposure and outcome variables. Results for some MR methods are absent because the harmonized data did not satisfy the quality check procedure, e.g., too few numbers of IVs.
