# Supplementary material for: The genetic architecture of multimodal human brain age
Source: Nat Commun. 2024 Mar 23;15:2604. doi: 10.1038/s41467-024-46796-6 (PMC10960798; doi:10.1038/s41467-024-46796-6)
Supplement: Supplementary file 5 — Reporting Summary [file 41467_2024_46796_MOESM5_ESM.pdf]

nature portfolio

Corresponding author(s): Junhao Wen

Last updated by author(s): Feb 8, 2024

Reporting Summary

Nature Portfolio wishes to improve the reproducibility of the work that we publish. This form provides structure for consistency and transparency in reporting. For further information on Nature Portfolio policies, see our Editorial Policies and the Editorial Policy Checklist.

Statistics

For all statistical analyses, confirm that the following items are present in the figure legend, table legend, main text, or Methods section.

n/a	Confirmed	
☐	☒	The exact sample size (*n*) for each experimental group/condition, given as a discrete number and unit of measurement
☐	☒	A statement on whether measurements were taken from distinct samples or whether the same sample was measured repeatedly
☐	☒	The statistical test(s) used AND whether they are one- or two-sided<br>*Only common tests should be described solely by name; describe more complex techniques in the Methods section.*
☐	☒	A description of all covariates tested
☐	☒	A description of any assumptions or corrections, such as tests of normality and adjustment for multiple comparisons
☐	☒	A full description of the statistical parameters including central tendency (e.g. means) or other basic estimates (e.g. regression coefficient) AND variation (e.g. standard deviation) or associated estimates of uncertainty (e.g. confidence intervals)
☐	☒	For null hypothesis testing, the test statistic (e.g. *F*, *t*, *r*) with confidence intervals, effect sizes, degrees of freedom and *P* value noted<br>*Give P values as exact values whenever suitable.*
☐	☒	For Bayesian analysis, information on the choice of priors and Markov chain Monte Carlo settings
☒	☐	For hierarchical and complex designs, identification of the appropriate level for tests and full reporting of outcomes
☐	☒	Estimates of effect sizes (e.g. Cohen's *d*, Pearson's *r*), indicating how they were calculated

Our web collection on statistics for biologists contains articles on many of the points above.

Software and code

Policy information about availability of computer code

Data collection

All data used in the current study are from the UK Biobank study. No software was used for data collection.

Data analysis

All data analyses used open-source software and package; some were developed by our team, and some were from other groups' development:

- MLNI: https://anbai106.github.io/mlni/, brain age prediction (v0.1.2)
- MEDICINE: https://labs.loni.usc.edu/medicine, knowledge portal for dissemination and GWAS summary statistics sharing
- MUSE: https://www.med.upenn.edu/cbica/sbia/muse.html, image preprocessing for GM-IDP (v0.0.1)
- PLINK: https://www.cog-genomics.org/plink/, GWAS and PRS (plink 2.0)
- FUMA: https://fuma.ctglab.nl/, gene mapping, genomic locus annotation (v1.5.0)
- GCTA: https://yanglab.westlake.edu.cn/software/gcta/#Overview, heritability estimates, and fastGWA (v1.94.1)
- LDSC: https://github.com/bulik/ldsc, genetic correlation, partitioned heritability, and heritability estimates (git version: aa33296)
- TwoSampleMR: https://mrcieu.github.io/TwoSampleMR/index.html, MR (v0.5.6)
- PRS-CS: https://github.com/getian107/PRScs, PRS (Aug 10, 2023)

For manuscripts utilizing custom algorithms or software that are central to the research but not yet described in published literature, software must be made available to editors and reviewers. We strongly encourage code deposition in a community repository (e.g. GitHub). See the Nature Portfolio guidelines for submitting code & software for further information.

Data

Policy information about availability of data

All manuscripts must include a data availability statement. This statement should provide the following information, where applicable:

- Accession codes, unique identifiers, or web links for publicly available datasets
- A description of any restrictions on data availability
- For clinical datasets or third party data, please ensure that the statement adheres to our policy

This study used the UK Biobank resource under Application Number 35148. No software was used for data collection. The GWAS summary statistics generated from our analyses are publicly available at the MEDICINE portal: https://labs.loni.usc.edu/medicine/organ_systems/brain. The raw imaging data are restricted to registered researchers and are protected and unavailable due to data privacy laws; access can be obtained at https://www.ukbiobank.ac.uk/. The gene-drug-disease network used data from the Drug Bank database (v.5.1.9: https://go.drugbank.com/) and the Therapeutic Target Database (updated by September 29th, 2021: https://idrblab.net/ttd/). Our genetic analyses also used GWAS summary statistics from the IEU OpenGWAS database (https://gwas.mrcieu.ac.uk/) and GWAS Catalog (https://www.ebi.ac.uk/gwas/).

Research involving human participants, their data, or biological material

Policy information about studies with human participants or human data. See also policy information about sex, gender (identity/presentation), and sexual orientation and race, ethnicity and racism.

Reporting on sex and gender	Throughout the manuscript, we consistently employed the variable of sex, incorporating both self-reported sex and genetic sex as covariates and quality-check criteria. Additionally, we conducted sex-stratified GWAS and brain age prediction models to investigate potential biases (sensitivity check)
Reporting on race, ethnicity, or other socially relevant groupings	The main three GWASs were specifically conducted for individuals of European ancestry, but we did perform several sensitivity check on non-European ancestry, etc.
Population characteristics	Study population is detailed in Method 1 in the main manuscript.
Recruitment	UK Biobank details the recruitment criteria at: https://www.ukbiobank.ac.uk/media/gnkeyh2q/study-rationale.pdf
Ethics oversight	University of Pennsylvania

Note that full information on the approval of the study protocol must also be provided in the manuscript.

Field-specific reporting

Please select the one below that is the best fit for your research. If you are not sure, read the appropriate sections before making your selection.

☒ Life sciences ☐ Behavioural & social sciences ☐ Ecological, evolutionary & environmental sciences

For a reference copy of the document with all sections, see nature.com/documents/nr-reporting-summary-flat.pdf

Life sciences study design

All studies must disclose on these points even when the disclosure is negative.

Sample size	No statistical tests were performed to determine the sample sizes. Sample sizes were decided by maximizing the imaging data after downloading from UKBB; sufficient quality check procedures for each MRI modalities were performed either by our team (T1-weighted MRI) or processed by the UK Biobank brain imaging team. The current sample size is the largest to date using the UK Biobank multimodal MRI data.
Data exclusions	All data exclusions during quality check is detailed in the Method 1 section in the main manuscript.
Replication	We checked the robustness of the main GWASs using the European populations via seven sensitivity analyses (not independent replication though). Overall, the primary GWASs were robust across sexes (female vs. male), random splits, imaging features (ROI vs. voxel-wise images), GWAS methods (linear vs. mixed linear model), and machine learning methods (Lasso regression vs. SVR. vs. CNN); however, their generalizability to non-European populations ($4646<N<5091$) and independent disease-specific populations (i.e., ADNI, N=1104) is limited potentially due to the small sample sizes (P-value is dependent on sample sizes). It's worth noting that their β values compared to the primary GWASs were significantly correlated: r=0.83 for ADNI and r=0.97-0.99 for the non-European populations. This highlights the importance of future studies including more diverse samples regarding disease populations and under-represented ethnic groups.
Randomization	No allocation is statistically determined. For training the brain age ML models, we randomly sub-sampled 500 (250 females) participants within each decade's range from 44 to 84 years old, resulting in the same 4000 participants for GM, WM, and FC-IDP. Another consideration is to explicitly exclude participants with common brain diseases, including mental and behavioral disorders (ICD-10 code: F; N=2678; Data-Field=41270) and diseases linked to the central nervous system (ICD-10 code: G group; N=3336). The motivation is to cover the entire age ranges in the UK Biobank study and with a relative large sample sized healthy populations (without common brain diseases) to train the machine learning models. After training the ML models, we applied the models to the rest of the populations.

	For all GWAS analyses, we controlled common covariates, such as age, sex, genetic PC, brain scan position, and total brain volume.
Blinding	The investigators were not blinded to group allocation throughout the experiments. The disease group labels were obtained from the UK Biobank using the ICD-10 code, but it is important to note that our study did not involve any clinical trials that would require blinding procedures. To train the brain age prediction ML models, we need to include participants without common brain diseases to model normal brain aging. This allows us to apply the trained model to the general populations to capture disease effects that drive the deviation from the typical brain aging trajectory.

Reporting for specific materials, systems and methods

We require information from authors about some types of materials, experimental systems and methods used in many studies. Here, indicate whether each material, system or method listed is relevant to your study. If you are not sure if a list item applies to your research, read the appropriate section before selecting a response.

Materials & experimental systems

n/a	Involved in the study
☒	☐ Antibodies
☒	☐ Eukaryotic cell lines
☒	☐ Palaeontology and archaeology
☒	☐ Animals and other organisms
☒	☐ Clinical data
☒	☐ Dual use research of concern
☒	☐ Plants

Methods

n/a	Involved in the study
☒	☐ ChIP-seq
☒	☐ Flow cytometry
☐	☒ MRI-based neuroimaging

Magnetic resonance imaging

Experimental design

Design type	NA
Design specifications	NA
Behavioral performance measures	NA

Acquisition

Imaging type(s)	Structural MRI (T1-weighted MRI); diffusion MRI; resting-state functional MRI
Field strength	3.5T
Sequence & imaging parameters	This can be found at the UK Biobank website for detail.
Area of acquisition	Brain

Diffusion MRI ☒ Used ☐ Not used

Parameters	Detailed parameters are publicly available on UKBB

Preprocessing

Preprocessing software	MUSE
Normalization	This is detailed at the original MUSE pipeline. https://pubmed.ncbi.nlm.nih.gov/26679328/
Normalization template	This is detailed at the original MUSE pipeline. https://pubmed.ncbi.nlm.nih.gov/26679328/
Noise and artifact removal	This is detailed at the original MUSE pipeline. https://pubmed.ncbi.nlm.nih.gov/26679328/
Volume censoring	This is detailed at the original MUSE pipeline. https://pubmed.ncbi.nlm.nih.gov/26679328/

Statistical modeling & inference

Model type and settings	Multivariate analyses (machine learning and deep learning) for deriving the three multimodal BAGs

Effect(s) tested | Beta coefficient are reported for GWAS, Odds ratio is reported for Mendelian randomization.

Specify type of analysis: ☐ Whole brain ☐ ROI-based ☒ Both

Anatomical location(s) | Using the MUSE atlas ROIs, and voxel-wise RAVESN for T1 MRI

Statistic type for inference | *Specify voxel-wise or cluster-wise and report all relevant parameters for cluster-wise methods.*

(See Eklund et al. 2016)

Correction | Bonferroni correction; we detailed all this in each result and method section.

Models & analysis

n/a	Involved in the study
☐	☒ Functional and/or effective connectivity
☒	☐ Graph analysis
☐	☒ Multivariate modeling or predictive analysis

Functional and/or effective connectivity | We used the UKBB processed functional connectivity measures downloeded from the website

Multivariate modeling and predictive analysis | MLNI package implemented several machine learning methods for brain age prediction
